# Hemorrhagic risk after intravenous thrombolysis for ischemic stroke in patients with cerebral microbleeds and white matter disease

**DOI:** 10.1007/s10072-020-04720-y

**Published:** 2020-09-29

**Authors:** Maria Luisa Capuana, Svetlana Lorenzano, Maria Chiara Caselli, Maurizio Paciaroni, Danilo Toni

**Affiliations:** 1 IRCCS Centro Neurolesi “Bonino Pulejo”, Palermo, Italy; 2 Emergency Department Stroke Unit, Department of Human Neurosciences, Policlinico Umberto I Hospital, Sapienza University of Rome, Viale del Policlinico, 155, 00161 Rome, Italy; 3 Department of Clinical and Sperimental Medicine, University of Pisa, Pisa, Italy; 4 Stroke Unit and Division of Internal and Cardiovascular Medicine, Santa Maria della Misericordia Hospital, University of Perugia, Perugia, Italy

**Keywords:** Cerebral microbleeds, Intravenous thrombolysis, Intracerebral hemorrhage, White matter disease, Outcome

## Abstract

**Objectives:**

Aim of this study was to evaluate the association between cerebral microbleeds (CMBs) and white matter disease (WMD) with intracerebral hemorrhage (ICH) after intravenous thrombolysis (IVT) with rt-PA. We also evaluated whether CMBs characteristics and WMD burden correlate with symptomatic ICH and outcome.

**Methods:**

We included acute ischemic stroke (AIS) patients treated with IVT. The number and location of CMBs as well as severity of WMD were rated analyzing pre- or post-treatment MRI. Multivariable regression analysis was used to determine the impact of CMB and WMD on ICH subgroups and outcome measures.

**Results:**

434 patients were included. CMBs were detected in 23.3% of them. ICH occurred in 34.7% of patients with CMBs. Independent predictors of parenchymal hemorrhage were the presence of CMBs (OR 2.724, 95% CI 1.360–5.464, *p* = 0.005) as well as cortical-subcortical stroke (OR 3.629, 95% CI 1.841–7.151, *p* < 0.001) and atherothrombotic stroke subtype (OR 3.381, 95% CI 1.335–8.566, *p* = 0.010). Either the presence, or number, and location of CMBs, as well as WMD, was not independently associated with the development of SICH. No independent association between the presence, number, or location of CMBs or WMD and outcome measures was observed.

**Conclusions:**

The results of our study suggest that the exclusion of eligible candidates to administration of IV rt-PA only on the basis of CMBs presence is not justified. The clinical decision should be weighed with a case-by-case approach. Additional data are needed to evaluate the benefit-risk profile of rt-PA in patients carrying numerous microbleeds.

**Electronic supplementary material:**

The online version of this article (10.1007/s10072-020-04720-y) contains supplementary material, which is available to authorized users.

## Background

Cerebral microbleeds (CMBs) are the expression of cerebral small vessel disease (SVD) and are commonly found in the elderly population, including patients with acute ischemic stroke (AIS) undergoing intravenous thrombolysis (IVT) [1]. Microbleeds can be discriminated from macrobleeds with specific sequences of magnetic resonance imaging (MRI) by various size cut-points. Typically, CMBs have a maximum diameter of 10 mm and appear larger on GRE sequences compared with the other tissue lesions because of the described “blooming effect” of the MR signal at the border of these lesions [2].

If CMBs represent a risk for the development of symptomatic intracerebral hemorrhage (SICH), or predict worse outcome after IVT in AIS, is a matter of debate. Another neuroimaging marker of SVD that could be in relation to the risk of ICH is white matter disease (WMD). Some evidence suggests that it can increase the risk of ICH during IVT but, so far, it does not represent an absolute exclusion criterion [3]. No clear leukoaraiosis volume threshold has been identified below in which no benefit or harm of IVT could be observed [4].

## Aims

The aim of this study was to evaluate the relationship between the presence and burden of CMBs and WMD with the development of ICH and SICH, and with clinical outcome after IVT in patients with AIS.

## Methods

### Study population and data collection

Patients with AIS receiving IVT according to the current guidelines [5] and undergoing magnetic resonance imaging (MRI) before or within 24 h after rt-PA infusion in two stroke centers were included in the study. We excluded patients for whom image quality was poor due to motion artifacts and patients treated with endovascular revascularization.

We collected demographic data (age, sex), baseline NIHSS score, systolic blood pressure and serum glucose levels, risk factors for stroke, pre-stroke therapies, etiopathogenetic subtypes, and neuroimaging variables.

### MRI protocol

Patients included in this study underwent cranial 1.5-T MRI. The imaging protocol consisted of T1-weighted imaging, T2-weighted imaging, T2*-weighted GRE imaging, fluid-attenuated inversion recovery (FLAIR), diffusion-weighted imaging (DWI), perfusion-weighted imaging (PWI), apparent diffusion coefficient (ADC), and 3-dimensional time-of-flight magnetic resonance angiography. We evaluated stroke characteristics, including vascular territory, and anatomical location. Micro-hemorrhages were identified according to a field guide of CMBs detection and interpretation [2]. CMB mimics were excluded. We recorded CMBs number, size (CMB < 5 mm, 5 mm > CMB < 10 mm), and their location: (1) lobar, if present in the cortex, subcortex, and white matter of the frontal, parietal, temporal, occipital, and insular lobes; (2) deep and infratentorial, if present in the head of the caudate, putamen, globus pallidus, internal capsule, thalamus, midbrain, pons, medulla, and cerebellum, according to The Brain Observer MicroBleeds Scale (BOMBS) [6]. WMD was defined as a hyperintense lesion on FLAIR and T2-weighted imaging, which was usually not seen or showed faint hypointensity on T1-weighted imaging. We rated WMD in the hemisphere contralateral to the index stroke using the scoring system presented by Fazekas et al. [7].

### Outcome measures

ICH was classified by using clinical and radiological criteria as follows: hemorrhagic infarction (HI, including HI-1 and HI-2) and parenchymal hemorrhage (PH, including PH-1 and PH-2), parenchymal remote hemorrhage (PHr1, PHr2) [8]. An ICH was defined as symptomatic (SICH) according to the SITS-MOST criteria in case of a local or remote PH-2 on the 22 to 36 h post-treatment imaging scan, combined with a neurological deterioration ≥ 4 points on the NIHSS score from baseline, or from the lowest NIHSS value between baseline and 24 h, or leading to death. [9]. Definitions of SICH according to NINDS (the National Institutes of Neurological Disorders and Stroke) [10] and ECASS (the European Cooperative Acute Stroke Study) [8] criteria were also used. Additional outcome measures were mortality at 7 days and mortality and functional dependency at 3 months. The latter was assessed by trained personnel using the modified Rankin Scale (mRS) score, by in-person or telephone interview [11], and was used as a dichotomous variable with mRS 3–6 defined as an unfavorable functional outcome.

### Statistical methods

We performed descriptive and univariate analyses for baseline clinical, demographic, and neuroimaging data, as well as outcome measures, comparing patients with and without CMBs. Percentages were calculated excluding missing cases. Continuous variables were expressed as mean (standard deviation, SD) or median (interquartile range, IQR) values based on their normal or non-normal distribution. Dichotomous variables between groups were compared by using chi-square or Fisher exact test, while Student’s *t* test or Mann-Whitney *U* test was used for continuous variables, depending on the normality of their distribution. Variables with univariate *p* value < 0.05 were included in the multivariable models. Multivariable logistic regression analyses were performed to identify variables independently associated with the risk of developing HI or PH and to evaluate whether CMBs and WMD were predictors of 90-day functional outcome and mortality at 7 days and 3 months. Statistical significance was set at a probability value of < 0.05. Data were analyzed using SPSS statistical software (IBM Corp, SPSS Statistics for Windows, Version 24, Armonk, NY).

## Results

In this study, we included 434 AIS patients (60.8% men; mean [SD] age 68.3 [13.5]) treated with IVT rt-PA 0.9 mg/kg and undergoing brain MRI over a period of approximately 5 years in two experienced stroke centers. We excluded 68 patients from the analysis because, given the presence of contraindications or particular clinical severity, they were unable to undergo MRI, they had poor MR image quality due to motion artifacts, or they received endovascular treatment.

Overall, the majority of patients (approximately 81%) underwent MRI after IVT within 24 h of stroke onset, while only in 19% of patients MRI was performed before IVT. The median time from rt-PA bolus to MRI was 1140 (IQR 384–1842) min (i.e., 19 h [IQR 6.4–30.7]) (Table 1). Demographics and baseline clinical and radiological characteristics of the whole cohort of patients and of the two subgroups defined by the presence/absence of CMBs are shown in Tables 1 and 2. Overall, CMBs were detected in 101 of 434 patients (23.3%). In these subjects, the mean (SD) number of CMBs was 2.45 (3.61), with a range from 1 to 27; the majority of patients had from 1 (59.4%) to 4 (30.7%) CMBs. CMBs were lobar in 73.3% of cases, deep/infratentorial in 41.6% of patients, and both lobar and deep/infratentorial in 14.9% of cases (Table 2). At univariate analysis, patients with CMBs were more likely to have a history of hypertension compared to those without (76.2% vs 62.5%, *p* = 0.011) (Table 1) and had more frequently a moderate to severe WMD (Fazekas Rating Scale score 2: 31.7% vs 16.5%, *p* = 0.001; Fazekas Rating Scale score 3: 13.9% vs 3.0%, *p* < 0.001); WMD was absent in a higher proportion of patients without CMBs (36.6% vs 17.8%, *p* < 0.001) (Table 2). The proportion of patients with a cortical-subcortical index stroke was 44.3% in patients with CMBs and 32.4% in those without CMB (*p* = 0.031) (Table 2).

**Table 1:** Baseline clinical characteristics of the whole cohort of patients and of the two subgroups identified by the presence/absence of CMBs

	All (*n* = 434)	CMBs (*n* = 101)	No CMBs (*n* = 333)	*p* value
Age (mean ± SD)	68.3 ± 13.5	69.0 (12.6)	68.1 (13.8)	0.517
Sex (males) (%)	264 (60.8)	66/101 (65.3)	198/333 (59.5)	0.288
Vascular risk factors				
Hypertension (%)	285/434 (65.7)	77/101 (76.2)	208/333 (62.5)	0.011
Diabetes mellitus (%)	74/434 (17.1)	15/101 (14.9)	59/333 (17.7)	0.502
Atrial fibrillation (%)	70/433 (16.2)	17/101 (16.8)	53/332 (16.0)	0.836
Current smoking (%)	82/429 (19.1)	18/100 (18.0)	64/329 (19.5)	0.746
Previous smoking (%)	64/419 (15.3)	16/97 (16.5)	48/322 (14.9)	0.703
Hypercholesterolemia (%)	138/434 (31.8)	30/101 (29.7)	108/333 (32.4)	0.606
Previous stroke within 3 months (%)	3/434 (0.7)	1/101 (1.0)	2/333 (0.6)	0.549
Previous stroke earlier than 3 months (%)	43/434 (9.9)	9/101 (8.9)	34/333 (10.2)	0.702
Previous diagnosis of TIA/amaurosis (%)	14/434 (3.2)	3/101 (3.0)	11/333 (3.3)	1.0
Congestive heart failure (%)	10/432 (2.3)	2/100 (2.0)	8/332 (2.4)	1.0
Medication history				
Prior antiplatelet therapy (%)	137/434 (31.6)	33/101 (32.7)	104/333 (31.2)	0.785
Prior oral anticoagulation (%)	16/415 (3.9)	7/98 (7.1)	9/317 (2.8)	0.069
Baseline NIHSS score, median (IQR)	8 (5–15)	9 (5–15)	8 (5–14)	0.682
Baseline systolic blood pressure (mm/hg) (mean ± SD)	146.8 ± 23.5	149.0 (24.9)	146.1 (23.1)	0.285
Baseline diastolic blood pressure (mmHg) (mean ± SD)	80.7 (13.8)	80.4 (14.6)	80.8 (13.5)	0.800
Baseline glucose level (mg/dl), median (IQR)	120.5 (104–149.25)	121.5 (107.25–150)	120.50 (104–147.25)	0.821
Onset-to-treatment time, median (IQR)	180 (135.0–222.25)	187.50 (145–229.25)	180 (130–220)	0.121
Door-to-needle time, median (IQR)	80.50 (55.0–116.25)	84 (55–110)	79 (53–118)	0.675
Bolus-MRI time, median (IQR)	1140 (384–1842)	1116 (363.50–2169.50)	1179 (388.50–1769.25)	0.914
Alteplase dose, mean (SD)	66.7 (13.2)	67.9 (62.75–74.25)	66.4 (13.5)	0.454
Stroke subtype (%)				
Atherothrombotic	34/393 (8.7)	4/92 (4.3)	30/301 (10.0)	0.093
Cardioembolic	130/393 (33.1)	32/92 (34.8)	98/301 (32.6)	0.691
Lacunar	45/393 (11.5)	9/92 (9.8)	36/301 (12.0)	0.566
Large vessel, other	39/393 (9.9)	7/92 (7.6)	32/301 (10.6)	0.396
Multiple/unknown	124/393 (31.6)	36/92 (39.1)	88/301 (29.2)	0.074
Unusual	21/393 (5.3)	4/92 (4.3)	17/301 (5.6)	0.794

*IQR*, interquartile range; *NIHSS*, National Institutes of Health Stroke Scale; *SD*, standard deviation; *TIA*, transient ischemic attack

**Table 2:** Radiological characteristics of the whole cohort of patients and of the two subgroups identified by presence/absence of CMBs

	All (*n* = 434)	CMBs (*n* = 101)	No CMBs (*n* = 333)	*p* value
Early ischemic sign at baseline neuroimaging (%)	44/343 (12.8)	13/85 (15.3)	31/258 (12.0)	0.433
Hyperdense MCA sign (%)	53/341 (15.5)	8/84 (9.5)	45/257 (17.5)	0.079
CMB parameters				
CMB number, mean (SD)	0.57 (2.02)	2.45 (3,61)	-	-
0 CMB (*n*, %)	333/434 (76.4)	-		
1 CMB (*n*, %)	60/434 (13.8)	60/101 (59.4)		
2–4 CMB (*n*, %)	31/434 (7.1)	31/101 (30.7)		
≥ 5 CMB (*n*, %)	10/434 (2.3)	10/101 (9.9)		
CMB localization	-		-	-
Lobar (%)	74/434 (17.1)	74/101 (73.3)		
Deep and infratentorial (%)	42/434 (9.7)	42/101 (41.6)		
Both lobar and deep/infratentorial (%)	15/434 (3.5)	15/101 (14.9)		
Fazekas WMD rating scale				
0 (%)	140/434 (32.3)	18/101 (17.8)	122/333 (36.6)	< 0.001
1 (%)	183/434 (42.2)	37/101 (36.6)	146/333 (43.8)	0.199
2 (%)	87/434 (20.0)	32/101 (31.7)	55/333 (16.5)	0.001
3 (%)	24/434 (5.5)	14/101 (13.9)	10/333 (3.0)	< 0.001
Stroke territory				
Anterior circulation (%)	288/423 (68.1)	70/97 (72.2)	218/326 (66.9)	0.326
Posterior circulation (%)	84/423 (19.9)	17/97 (17.5)	67/326 (20.6)	0.512
Both anterior and posterior (%)	51/423 (12.1)	10/97 (10.3)	41/326 (12.6)	0.547
Stroke localization				
Cortical (%)	141/421 (33.5)	25/97 (25.8)	116/324 (35.8)	0.066
Subcortical (%)	132/421 (31.4)	29/97 (29.9)	103/324 (31.8)	0.724
Cortical-subcortical (%)	148/421 (35.2)	43/97 (44.3)	105/324 (32.4)	0.031
ICH (All) (%)	107/432 (24.8)	35/101 (34.7)	72/331 (21.8)	0.009
HI (%)	52/432 (12.0)	12/101 (11.9)	40/331 (12.1)	0.956
PH (%)	49/432 (11.3)	19/101 (18.8)	30/331 (9.1)	0.007
PHr* (%)	6/432 (1.4)	4/101 (4.0)	2/331 (0.6)	0.029

*Of the 6 PHr, 4 had also PH and 2 also HI

*CMB*, cerebral microbleeds; *HI*, hemorrhagic infarction; *ICH*, intracerebral hemorrhage; *MCA*, middle cerebral artery; *NIHSS*, National Institutes of Health Stroke Scale; *PH*, parenchymal hemorrhage; *PHr*, remote parenchymal hemorrhage; *SD*, standard deviation, *WMD*, white matter disease

Overall, ICH (all types) were detected in 107 (24.8%) patients of the whole cohort, and more frequently in patients with CMBs (34.7%) than in those without CMBs (21.8%) (*p* = 0.009), particularly severe ICH (PH: 18.8% vs 9.1%, *p* = 0.007; PHr: 4.0% vs 0.6%, *p* = 0.029) (Table 2). HT resulted in symptomatic as per the SITS-MOST definition in 2 (0.5%) patients (with both PH and PHr) (Table 3). SICH as per any definition was more likely to develop in patients with CMBs (SICH/SITS-MOST, 2.0% vs 0, *p* = 0.054; SICH/NINDS, 12.9% vs 6.2%, *p* = 0.041; SICH/ECASS, 8.3% vs 2.2%, *p* = 0.015) (Table 3).

**Table 3:** Outcome measures of the whole cohort of patients and of the two subgroups identified by the presence/absence of CMBs

	All (*n* = 434)	CMBs (*n* = 101)	No CMBs (*n* = 333)	*p* value
Death at 7 days (%)	9/352 (2.6)	2/67 (3.0)	7/285 (2.5)	0.682
mRS 3–6 at 3 months (%)	130/388 (33.5)	39/91 (42.9)	91/297 (30.6)	0.031
Death at 3 months (%)	33/389 (8.5)	11/92 (12.0)	22/297 (7.4)	0.171
SICH/SITS-MOST (%)	2/432(0.5%)	2/101 (2.0)	0	0.054
SICH/NINDS (%)	28/361 (7.8)	11/85 (12.9)	17/276 (6.2)	0.041
SICH/ECASS (%)	13/360 (3.6)	7/84 (8.3)	6/276 (2.2)	0.015

*mRS*, modified Rankin scale, *SICH*, symptomatic intracranial hemorrhage

**Fig. 1:**
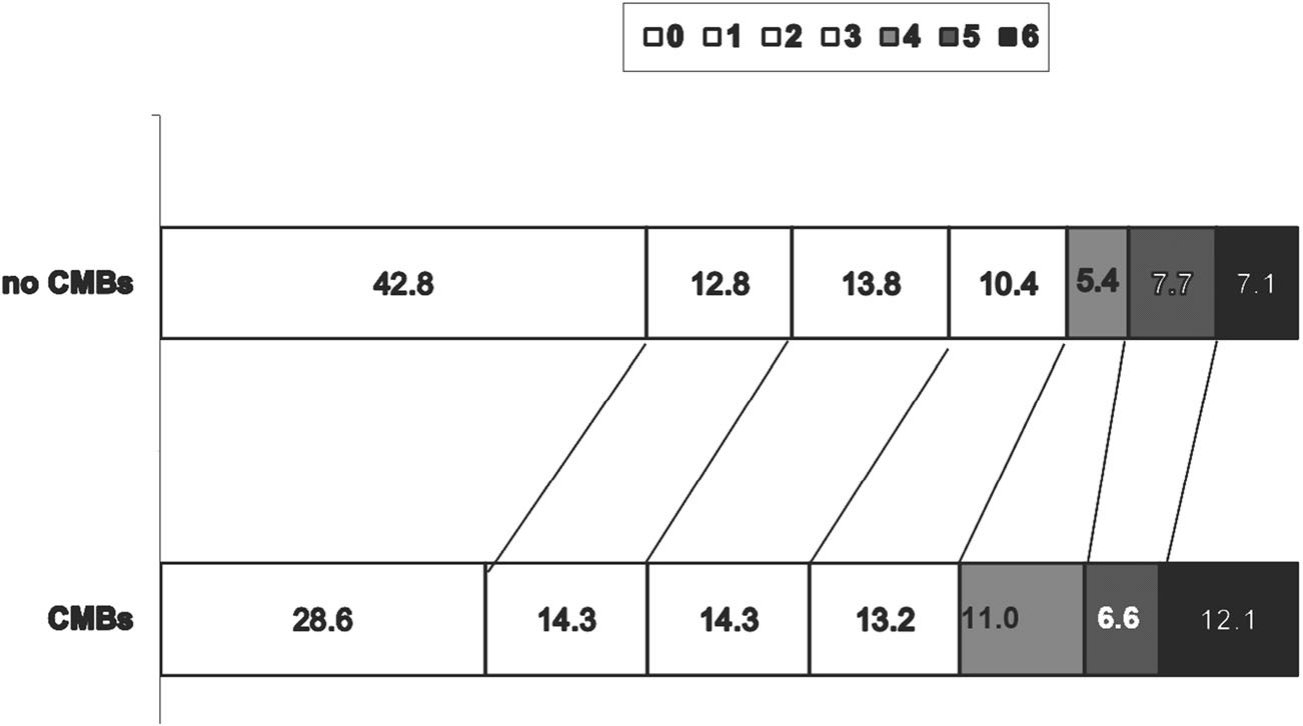
Distribution of mRS score in patients without and with CMBs

Mortality, both at 7 days and 90 days, did not significantly differ between patients with or without CMBs. Conversely, patients with CMBs were found to have a worse functional outcome (mRS 3–6) at 90 days more frequently compared with patients without CMBs (42.9% vs 30.6%, *p* = 0.031). Figure 1 reports the distribution of mRS scores in patients without and with CMBs.

Compared with those without any type of ICH, the mean number of CMBs was significantly higher in patients with any ICH (0.98 vs 0.44, *p* = 0.006), PH (0.69 vs 0.56, *p* = 0.008), PHr (8.33 vs 0.46, *p* = 0.003), and SICH by any definition (SICH/SITS-MOST: 11.0 vs 0.52, *p* = 0.001; SICH/NINDS: 1.39 vs 0.52, *p* = 0.022; SICH/ECASS: 2.46 vs 0.52, *p* = 0.004). A higher burden of CMBs was found more frequently in patients with any ICH (CMBs 2–4: 13.1% vs 5.2%, *p* = 0.006), PH (at least 1 CMB: 22.4% vs 12.0%, *p* = 0.042; with a trend toward the statistical significance for CMBs 2–4: 14.3% vs 6.3%, *p* = 0.069), PHr (CMBs ≥ 5: 33.3% [2/6 subjects] vs 1.9% [8/426], *p* = 0.007), SICH/SITS-MOST (CMBs 2–4: 50% [1/2 subjects] vs 7.0% [30/430], *p* = 0.019; CMBs ≥ 5: 50% [1/2 subjects] vs 2.1% [9/430], *p* = 0.046), SICH/NINDS (CMBs 2-4: 17.9% vs 6.3%, *p* = 0.040), and SICH/ECASS (CMBs ≥ 5: 15.4% vs 1.7%, *p* = 0.030).

Furthermore, the mean number of CMBs was higher in patients with unfavorable functional outcome (0.72 vs 0.53, *p* = 0.040) and at least 1 CMB was found more frequently in these patients compared with those who reached a favorable outcome (19.2% vs 10.1%, *p* = 0.012).

Deep/infratentorial location of CMBs resulted significantly associated with any ICH (16.8% vs 7.4% in patients without ICH, *p* = 0.004), PH (20.4% vs 8.4%, *p* = 0.017), while lobar CMBs were more frequently found in patients with PHr (66.7% [4/6 subjects] vs 16.4% [70/426], *p* = 0.009), SICH/SITS-MOST (100.0% [2/2 subjects] vs 16.7% [72/430], *p* = 0.029), and SICH/ECASS (46.2% vs 16.4%, *p* = 0.014), with only a trend toward the statistical significance for SICH/NINDS (32.1% vs 16.5%, *p* = 0.066).

Conversely, CMBs location seems not to have any correlation with mortality or functional outcome.

Regarding WMD, a higher burden of this radiological marker of cerebral SVD was significantly associated only with more severe ICH, i.e., PHr (Fazekas Rating Scale score 3: 33.3% [2/6 subjects] vs 5.2% [22/426], *p* = 0.039) (but not with SICH) and with mRS 3–6 at 90 days (Fazekas Rating Scale score 2: 26.2% vs 17.1% in patients with 90-day mRS 0–2, *p* = 0.035).

Multivariable regression analyses for outcome measures are reported in Tables 4 and 5. As regards PH, after adjustment for the index stroke anatomical location (subcortical, cortical-subcortical), presence of CMBs, number of CMBs as categorical variable, location of CMBs (deep/infrantentorial vs lobar), and atherothrombotic stroke subtype, the presence of CMBs independently predicted PH (OR 2.724, 95% CI 1.360–5.464, *p* = 0.005) (Table 4, section B). When the number of CMBs was included as a continuous variable in the model, the deep/infratentorial location of CMBs (OR 3.121, 95% CI 1.342–7.259, *p* = 0.008) resulted in an independent predictor of PH (Table 4, section B). Given the small numbers, no multivariable analysis was performed for PHr and SICH, except for SICH/NINDS definition which either the presence or the number of CMBs as well as their location or WMD did not result independently associated with (Table 4, section C).

**Table 4:** Multivariable analyses for radiological outcome measures (HI, PH, SICH/NINDS)

	OR	95% CI	*p*
A. Multivariable analysis for HI*			
Cortical-subcortical stroke	2.957	1.508–5.797	0.002
Both anterior and posterior stroke	4.142	1.804–9.509	0.001
Cardioembolic stroke	2.337	1.229–4.443	0.010
WMD (absence)	2.299	1.196–4.422	0.013
B. Multivariable analyses for PH			
First model**			
Cortical-subcortical stroke	3.629	1.841–7.151	< 0.001
CMB (absence vs presence)	2.724	1.360–5.464	0.005
Atherothrombotic stroke	3.381	1.335–8.566	0.010
Second model***			
Cortical-subcortical stroke	3.804	1.934–7.481	< 0.001
Deep/infratentorial location of CMB	3.121	1.342–7.259	0.008
Atherothrombotic stroke	3.118	1.244–7.817	0.015
C. Multivariable analysis for SICH/NINDS****			
NIHSS at baseline	1.119	1.050–1.193	0.001
Cortical-subcortical stroke	3.123	1.265–7.708	0.013

*Adjusted for the history of diabetes mellitus, cortical-subcortical stroke, both anterior and posterior stroke, absence/presence of WMD at the modified Fazekas Rating Scale, cardioembolic stroke

**Adjusted for subcortical stroke, cortical-subcortical stroke, CMB location (deep/infratentorial vs lobar), CMB absence vs presence, CMB number as categorical variable, atherothrombotic stroke

***Adjusted for subcortical stroke, cortical-subcortical stroke, CMB location (deep/infratentorial vs lobar), CMB number as a continuous variable, atherothrombotic stroke

****Adjusted for NIHSS at baseline, cortical-subcortical stroke, hyperdense MCA sign, presence of CMBs, number of CMBs

**Table 5:** Multivariable analyses for clinical outcome measures

	OR	95% CI	*p*
A. Death at 7 days*			
NIHSS at baseline	1.155	1.033–1.291	0.012
B. mRS 3–6 at 3 months**			
NIHSS at baseline	1.302	1.185–1.431	< 0.001
Diabetes	4.680	1.487–14.726	0.008
Cortical-subcortical stroke	3.684	1.247–10.880	0.018
C. Death at 3 months***			
NIHSS at baseline	1.121	1.052–1.196	< 0.001
Age	1.091	1.039–1.146	0.001
Diabetes	3.080	1.223–7.755	0.017
Atherothrombotic stroke	4.978	1.629–15.212	0.005

*Adjusted for Age, NIHSS at baseline, atherothrombotic stroke subtype

**Adjusted for age, history of diabetes, past smoking, NIHSS at baseline, alteplase dose, blood glucose levels at baseline, atherothrombotic and lacunar stroke subtypes, cortical and cortical-subcortical strokes, posterior stroke, moderate WMD (modified Fazekas Scale equals to 2), presence/absence of CMBs, CMB number

***Adjusted for age, NIHSS at baseline, history of diabetes, history of AF, atherothrombotic stroke subtype, cortical-subcortical stroke

No independent association between the presence, number, or location of CMBs or WMD and either intra-hospital or 90-day mortality as well as a functional outcome at 90 days was observed (Table 5).

The results of multivariate analyses did not change overall after the addition of time interval variables (i.e., onset-to-treatment time, door-to-needle time, and bolus-MRI time) to the models (see e-Tables in the supplemental material).

## Discussion

The objective of this study was to evaluate whether the presence of radiological markers of cerebral SVD could be a possible limitation to the use of IVT. We found that although the presence of CMBs and their deep/infratentorial location independently predicted the development of large hemorrhagic transformation (PH), they were not related either to SICH or unfavorable functional outcome.

In the last years, many research papers have been conducted on this topic. The prevalence of CMBs observed in our study cohort (23.3%) was in keeping with that reported in the literature (range 15 to 38%) [12, 13]. Recently, some meta-analyses focusing on clinical outcomes have been also published.

A metanalysis [14], including 9 studies comprising 2479 patients with AIS receiving IVT, found that high CMB burden (> 10 CMBs) on pre-treatment MRI was independently associated with SICH. These observations suggest that CMB burden may be included in individual risk stratification predicting the risk of SICH after treatment.

More recently, in a multicenter study analyzing 672 patients [15], the authors concluded that there is an increased risk of SICH after IVT when more than 10 CMBs are present. Chacon-Portillo et al. [16] obtained similar results as regards CMB burden while they did not find any association between CMB presence or location with SICH.

Similarly to our study, the last recently published large meta-analysis [13] reported that patients with CMBs had increased risk of PH and PHr but not of SICH. Concerning outcome measures, five or more and > 10 CMBs independently predicted poor outcome; conversely, in our cohort, either the presence or the burden of CMBs as well as their location did not result in independent predictors of 90-day mortality and unfavorable functional outcome in adjusted analyses.

In all these studies, CMB burden is the parameter that seems to be strongly associated with the risk of ICH; of note, the number of patients with high CMB burden (CMBs > 10) was very low. Results regarding clinical outcome are conflicting.

In our study, despite the fact that patients with CMBs and, among these, those with higher mean number of CMBs were more likely to develop any type of ICH (except for the milder one, i.e., HI), whether symptomatic or not, it was evident that presence, burden, or location of CMBs did not have any impact on either intra-hospital or 90-day mortality as well as on functional outcome at 90 days. Similarly, despite the significant univariate association between the higher burden of WMD and severe ICH (i.e., PH) and 90-day unfavorable outcome, WMD did not result in an independent predictor of SICH, mortality, or functional outcome in adjusted analyses. Therefore, in our cohort, patients with moderate to severe leukoaraiosis appeared to still have clinical benefit from IV rt-PA, although an increased risk of symptomatic ICH in these patients is described [4]. NIHSS at baseline is the parameter that strongly correlates with clinical outcome in our study.

As regards the location of CMBs, in our study, their position in deep/infratentorial brain areas resulted in an independent predictor of PH. Data analysis showed that CMBs were lobar in the majority of our patients. In the literature, it is reported that more than 87% of CMBs located in the lobar regions and predominantly in the posterior cortex, are likely caused by cerebral amyloid angiopathy (CAA). On the other hand, hypertensive microbleeds predominantly develop in the deep gray matter of the brainstem [17]. No multivariable analysis was performed for PHr, due to the small numbers, but most patients with PHr had CMBs and, in particular, lobar CMBs.

Our study has limitations. First, given the retrospective design and the objectives of this study, patients were not consecutively included because only patients who underwent MRI within approximately 24 h were taken into consideration. However, the percentage of patients (around 15%) excluded from the analysis is relatively small and overall acceptable. Second, only few patients underwent MR before thrombolysis, and hence, for CMB and WMD assessment, we used the follow-up MRI which, based on our local protocol, is usually performed within 24 h from symptoms onset. Although studies are still limited, a possible appearance of new CMBs in the first 24 h after thrombolysis has been described [18]. However, even hypothesizing a post-IVT increase of CMBs in our series of patients, this would have increased rather than reduced the possible relationship between CMBs and SICH or unfavorable outcomes.

Because of the small number of pre-IVT MRI in our patient cohort and since we did not systematically collect data on the exact location of hemorrhagic transformation, we were not able to evaluate, for those patients who developed a local hemorrhagic transformation of the index infarct and/or a remote ICH after IVT, whether there was a relationship between the specific location of PH and PHr and previous presence of CMBs or WMD in the same site. This can be considered likely particularly for CMBs, also based on available data from population-based studies which found a spatial colocalization between the site of CMB and the region of hemorrhagic stroke [19].

However, these aspects should be thoroughly investigated in large prospective studies on ischemic stroke patients receiving acute reperfusion/revascularization treatments.

Another potential limitation of our study, similarly to all studies on CMBs, is that patients undergoing a good-quality MRI (in contrast to computed tomography) are usually less severely ill and more collaborative, which may represent a potential selection bias.

Finally, the small number of SICHs lowers statistical power. Assessment of the association of the individual number of CMBs with bleeding risk was complicated by the low rate of patients with a large number of CMBs (CMBs > 10). Therefore, in our cohort conclusions, concerning the rare patients with a great burden of CMBs cannot be drawn. For this reason, further analyses in larger studies taking into account CMBs number and location, and their relationship with measures of functional outcome, are needed.

Current evidence points out that patients with CMBs should not be ruled out from a potential beneficial therapy as IVT. In the absence of comparative data demonstrating lack of functional benefit from IVT in AIS patients with CMBs vs those without, all available data do not justify the exclusion of eligible candidates to administration of IV rt-PA on the basis of CMB presence in case MRI, besides CT scan or alone, is performed in the acute setting.

## Electronic supplementary material

ESM 1 (DOC 32 kb)

## Data Availability

Not applicable.
