# Supplementary material for: Hemorrhagic risk after intravenous thrombolysis for ischemic stroke in patients with cerebral microbleeds and white matter disease
Source: Neurol Sci. 2020 Sep 29;42(5):1969–76. doi: 10.1007/s10072-020-04720-y (PMC8043883; doi:10.1007/s10072-020-04720-y)
Supplement: Supplementary file 1 — (DOC 32 kb) [file 10072_2020_4720_MOESM1_ESM.doc]

**Table 4. Multivariable analyses for radiological outcome measures (HI, PH, SICH/NINDS) after addition of time intervals to the models**

**A. Multivariable analysis for HI***

|  | **OR** | **95% CI** | **p** |
| --- | --- | --- | --- |
| Cortical-subcortical stroke | 4.464 | 1.844-10.807 | 0.001 |
| Both anterior and posterior stroke | 9.398 | 3.200-27.601 | <0.001 |
| Cardioembolic stroke | 3.250 | 1.468-7.195 | 0.004 |
| WMD (absence) | 2.317 | 1.024-5.244 | 0.044 |
| Diabetes mellitus | 2.680 | 1.097-6.549 | 0.031 |
| Onset-to-treatment time | 0.993 | 0.986-0.999 | 0.025 |

*Adjusted for history of diabetes mellitus, cortical-subcortical stroke, both anterior and posterior stroke, absence/presence of WMD at the modified Fazekas rating scale, cardioembolic stroke, onset-to-treatment time, door-to-needle time, bolus-MRI time.

**B. Multivariable analyses for PH**

|  | **OR** | **95% CI** | **P** |
| --- | --- | --- | --- |
| **First model*** |  |  |  |
| Cortical-subcortical stroke | 4.109 | 1.812-9.321 | 0.001 |
| Deep/infratentorial location of CMB | 4.330 | 1.628-11.521 | 0.003 |

* Adjusted for subcortical stroke, cortical-subcortical stroke, CMB location (deep/infratentorial vs lobar), CMB absence vs presence, CMB number as categorical variable, atherothrombotic stroke, onset-to-treatment time, door-to-needle time, bolus-MRI time.

|  | **OR** | **95% CI** | **p** |
| --- | --- | --- | --- |
| **Second model*** |  |  |  |
| Cortical-subcortical stroke | 4.109 | 1.812-9.321 | 0.001 |
| Deep/infratentorial location of CMB | 4.330 | 1.628-11.521 | 0.003 |

*Adjusted for subcortical stroke, cortical-subcortical stroke, CMB location (deep/infratentorial vs lobar), CMB number as continuous variable, atherothrombotic stroke, onset-to-treatment time, door-to-needle time, bolus-MRI time.

**C. Multivariable analysis for SICH/NINDS***

|  | **OR** | **95% CI** | **p** |
| --- | --- | --- | --- |
| Cortical-subcortical stroke | 8.667 | 2.393-31.386 | 0.001 |

*Adjusted for NIHSS at baseline, cortical-subcortical stroke, hyperdense MCA sign, presence of CMBs, number of CMBs, onset-to-treatment time, door-to-needle time, bolus-MRI time.

**Table 5. Multivariable analyses for clinical outcome measures after addition of time intervals to the models**

**A. Death at 7 days***

|  | **OR** | **95% CI** | **p** |
| --- | --- | --- | --- |
| NIHSS at baseline | 1.144 | 0.992-1.320 | 0.064 |
| Atherothrombotic stroke | 9.456 | 1.293-69.139 | 0.027 |

*Adjusted for Age, NIHSS at baseline, atherothrombotic stroke subtype, onset-to-treatment time, door-to-needle time, bolus-MRI time.

**B. mRS 3-6 at 3 months***

|  | **OR** | **95% CI** | **p** |
| --- | --- | --- | --- |
| NIHSS at baseline | 1.460 | 1.237-1.724 | <0.001 |
| Age | 1.083 | 1.012-1.159 | 0.021 |
| Diabetes | 21.283 | 2.436-185.954 | 0.006 |
| Baseline blood glucose levels | 0.979 | 0.960-0.999 | 0.042 |

*Adjusted for age, history of diabetes, past smoking, NIHSS at baseline, alteplase dose, blood glucose levels at baseline, atherothrombotic and lacunar stroke subtypes, cortical and cortical-subcortical strokes, posterior stroke, moderate WMD (modified Fazekas scale equals to 2), presence/absence of CMBs, CMB number, onset-to-treatment time, door-to-needle time, bolus-MRI time.

**C. Death at 3 months***

|  | **OR** | **95% CI** | **p** |
| --- | --- | --- | --- |
| NIHSS at baseline | 1.157 | 1.065-1.258 | 0.001 |
| Age | 1.066 | 1.005-1.131 | 0.034 |
| Diabetes | 5.577 | 1.726-18.019 | 0.004 |
| Atherothrombotic stroke | 5.141 | 1.150-22.989 | 0.032 |

*Adjusted for age, NIHSS at baseline, history of diabetes, history of AF, atherothrombotic stroke subtype, cortical-subcortical stroke, onset-to-treatment time, door-to-needle time, bolus-MRI time.
